# Regulation of PBP2x Surface Localization by *aliD* and *clpL* Alters β-Lactam Resistance in Pneumococcus

**DOI:** 10.3390/pathogens15070738

**Published:** 2026-07-14

**Authors:** Lucas R. G. Crosby, Md Fahim Khan, Larry S. McDaniel, Lance E. Keller

**Affiliations:** 1 Department of Cell and Molecular Biology, University of Mississippi Medical Center, Jackson, MS 39216, USA; lcrosby@umc.edu (L.R.G.C.);; 2 Center for Immunology and Microbial Research, University of Mississippi Medical Center, Jackson, MS 39216, USA

**Keywords:** *aliD*, *clpL*, PBP2x, *Streptococcus pneumoniae*

## Abstract

*Streptococcus pneumoniae* is a leading cause of respiratory infections and is often treated with β-lactam antibiotics despite frequent resistance. The β-lactam resistance is through mutations in penicillin-binding proteins (PBPs), but the impact of PBP regulation remains poorly understood. Here, we investigate the role of gene regulation by the oligopeptide-binding protein *aliD* on β-lactam susceptibility. *aliD*-expressing strains exhibit elevated minimum inhibitory concentrations (MICs) to the β-lactam antibiotics amoxicillin and cefdinir compared to isogenic *aliD* mutants. In contrast, *aliD* had minimal effects on susceptibility to vancomycin, suggesting a mechanism specific to β-lactam antibiotics. We demonstrate that *aliD* increases *clpL* expression with or without antibiotics present. Inducible expression of *clpL* resulted in stepwise increases in amoxicillin MIC, demonstrating that elevated *clpL* expression directly contributes to decreased β-lactam susceptibility. Furthermore, we show that increased *clpL* expression enhances PBP2x surface exposure in a dose-dependent manner. Additionally, *aliD*-expressing strains exhibited significantly increased cell wall cross-linking relative to *aliD* mutants. Together, these findings identify a novel regulatory pathway in which *aliD* enhances *clpL* expression, promotes PBP2x surface exposure, and increases peptidoglycan cross-linking, ultimately reducing β-lactam susceptibility. This work expands current understanding of pneumococcal antibiotic resistance and suggests that peptide sensing through oligopeptide transport systems may influence antibiotic susceptibility in host-specific environments.

## 1. Introduction

*Streptococcus pneumoniae*, also known as the pneumococcus, is the leading cause of lower respiratory tract infections (LRTIs) [1], which include pneumonia and bronchitis. Annually, *S. pneumoniae* causes almost 50% (~1,100,000 out of 2,300,000) of deaths associated with LRTIs, with the highest mortality rate being in children under the age of five [2]. The pneumococcus accounts for more deaths than the next three most common causes of LRTIs combined: *Haemophilus influenzae*, influenza, and respiratory syncytial virus [3].

This high mortality is due to a number of *S. pneumoniae* virulence factors, such as the highly variable pneumococcal capsule, PspA, and pneumolysin [4]. Clinicians use antibiotics and vaccines to treat and prevent *S. pneumoniae* infections. The advent of the pneumococcal vaccine is one of the most effective tools used to combat pneumococcal infections. Current vaccines target a subset of the more than 107 pneumococcal capsules currently known [5]. Each capsule serotype has an antigenically distinct carbohydrate structure that produces no cross-reactive antibodies to different serotypes. Current vaccines contain the most clinically relevant serotypes, as creating a vaccine targeting all serotypes would not be practical.

Pneumococci-expressing serotypes included within the vaccine are subjected to heightened immunological pressure. Conversely, pneumococcal strains expressing non-vaccine serotypes remain unimpeded by this immunological constraint. Consequently, the immunological landscape sculpted by vaccination preferentially facilitates the proliferation of non-vaccine serotypes, a phenomenon termed serotype replacement [6]. This shift permits the ascendance of previously low-prevalence serotypes, which may now exploit the vacated ecological niche to establish colonization and lead to disease development. This epidemiological observation is further increased through strain-level serotype switching [7]. Serotype switching refers to the change from one pneumococcal serotype to another through genetic exchange. Both serotype switching and replacement have limited the effectiveness of pneumococcal vaccination.

This selective pressure changes the epidemiology of pneumococcal strains present in the population. A notable change in the pneumococcal population is the rise of nonencapsulated *S. pneumoniae* (NESp). NESp lack a capsule and are divided into either group 1 or group 2 NESp. Group 1 NESp contain capsular polysaccharide synthesis (*cps*) genes but are not functional due to mutations. Group 2 NESp contain novel genes in the *cps* locus not related to the production of capsules. Group 2 is further divided into null capsule clades (NCCs), dependent on the genes present. NCC1 contains the surface protein *pspK*, while NCC2 and NCC3 contain *aliC* and *aliD* or only *aliD,* respectively [8]. The *aliC* and *aliD* genes encode substrate-binding proteins (SBP) used for oligopeptide importation through the Ami oligopeptide transport system [9,10]. The Ami system was the first oligopeptide permease discovered in Gram-positive bacteria and plays an important role in bacterial metabolism, as it directly imports various polypeptides and polyamines [10]. Oligopeptide transporters utilize a surface-bound SBP that binds exogenous compounds and imports them through an ABC transporter. This importation into the cell also impacts gene expression in multiple pathways, such as the metabolism of specific carbohydrates, cell signaling, competence, and colonization factors [11,12]. All pneumococci contain the SBPs AmiA, AliA, and aliB, while NESp can contain the additional *aliC* and *aliD* SBPs. Interestingly, while *aliD* is typically found only in NESp strains, there are three encapsulated serotypes that also express *aliD*: 25A, 25F, and 38. Of note is serotype 38, which has been increasing in prevalence in certain populations [13]. SBPs have a broad range of substrate specificity with overlapping substrates along with unique substrates. Changes in gene expression depend on the SBP and the substrate being imported [14].

Selective pressure against specific populations has also changed the prevalence of antibiotic resistance [15]. Antibiotic resistance generally occurs in one of two ways: extrinsic or intrinsic acquisition. Extrinsic is the acquisition of a new gene to perform a specific function, such as using efflux pumps to directly remove antibiotics from the cell or utilizing proteins like β-lactamases to inactivate antibiotics. For intrinsic acquisition, the bacteria can experience mutational resistance that modifies bacterial proteins, such as gyrase or topoisomerase mutations, leading to quinolone resistance [16]. Additionally, changes in gene expression levels can impact antibiotic susceptibility [17]. Since oligopeptide transporters can impact gene expression, adding additional SBPs—as seen in NESp and certain serotypes—may change antibiotic susceptibility.

Previous work examined the role of *aliD* on gene regulation and the impact on bacterial virulence. One of the genes regulated by the oligopeptide transporter *aliD* is the chaperone *clpL* [9]. However, the possible interaction between *aliD* and ClpL has not been explored. ClpL is an HSP100 chaperone protein in the caseinolytic protease system. Its most well-known function is assisting in protein refolding due to incorrect conformation or trafficking proteins to the ClpP protease for degradation. An additional function of ClpL is the transportation of penicillin-binding protein 2x (PBP2x) to the cell surface [18].

Penicillin-binding proteins (PBPs) are integral to the creation of the cell wall. The bacterial cell wall is formed from a mesh-like lattice of peptidoglycan. PBPs form cross-links in the peptidoglycan stands to give structure and stability to the cell wall. All strains of *S. pneumoniae* contain six PBPs: 1a, 1b, 2x, 2a, 2b, and 3 [19]. PBPs are the target for β-lactam antibiotics, which contain a β-lactam ring that will allosterically bind to the PBP, resulting in irreversible loss of function [19] and an inability of the bacteria to form cross-links between the peptidoglycan strands. This will destabilize the cell wall and render the bacteria vulnerable to outside threats such as osmotic pressure, leading to cell death. The primary mechanism for β-lactam resistance in *S. pneumoniae* is via mutations to the PBPs that inhibit β-lactam binding and can be transferred to other strains to increase rates of resistance [19]. This differs from many bacteria that employ β-lactamases to break the β-lactam ring [19,20]. In the last decade, rates of antibiotic resistance have varied, with resistance to some antibiotics increasing while others have decreased [21].

It is important to have a complete understanding of resistance mechanisms to more effectively combat changes in antibiotic resistance. Due to the role of PBPs on β-lactam resistance and the regulation of *clpL* expression by *aliD*, this study aimed to further characterize the relationship between *aliD* and antibiotic susceptibility. Additionally, this study examined how these pneumococcal regulatory mechanisms may contribute to the fitness, survival, and potential antibiotic resistance of the bacteria. Through analysis of antibiotic minimum inhibitory concentrations (MICs), gene expression, and cell wall composition, the following study provides insight into how altering *clpL* expression impacts β-lactam antibiotic susceptibility through altered cell wall cross-linking mediated by PBP2x.

## 2. Materials and Methods

### 2.1. Bacterial Strains and Growth Conditions

*Streptococcus pneumoniae* strains used in the current study are detailed in Table 1. All pneumococci were grown at 37 °C with 5% CO_2_ in Todd Hewitt broth supplemented with 0.5% yeast extract (THY) or on Columbia blood agar supplemented with 5% defibrinated sheep blood (BA). Both sourced from ThermoFisher, Waltham, MA, USA. Selection with antibiotics was done where appropriate, and concentrations are indicated on Table 1.

### 2.2. Strain Creation

Transformation of constructs followed the protocol described in Thompson et al. [26]. All constructs were created using GoldenGate assembly, and primers used for strain construction are detailed in Table 2, with restriction sites underlined and italicized. LRC1 was created by transforming R36A with the LacI and TetR repressor downstream of the *prs1* locus amplified from strain PLT2-2 using KLO_448 and KLO_449 [27]. ClpL deletion was made through allelic replacement of DNA containing *clpL* flanking regions amplified from R36A gDNA (KLO_426 and KLO_427; KLO_430 and KLO_431) and a spectinomycin marker amplified (KLO_428 and KLO_429) from the plasmid pPEPX. Complementation of inducible *clpL* was created by inserting the *clpL* gene (amplified with KLO_256 and KLO_257) into the linearized pPEPY-Plac vector (KLO_254 and KLO_255) and transforming into R36A. For LRC2, PBP2x tagged with SmBiT was created through amplifying the flanking region from R36A gDNA (KLO_201 and KLO_202; KLO_205 and KLO_206), and erm resistance marker (KLO_203 and KLO_204) from pPEPZ was added to LRC1 [28]. This created a SmBiT tag, which was used as the template to create the HiBiT-tagged PBP2x using primer pair KLO_201 and KLO_362 along with KLO_206 and KLO_363. The resulting fragments were ligated and transformed into LRC1, creating LRC2. Construct-containing ClpL-luciferase transcriptional fusion was created through amplification of flanking region (KLO_280 and KLO_281; KLO_286 and KLO_287), luciferase gene (KLO_282 and KLO_283) from PEP1-LGZ [29], and kanamycin marker (KLO_284 and KLO_285) from pPEPY [28]. LRC2 was created through amplifying the PBP2x-HiBiT fusion from LRC5 and transformed into LRC4. LRC4 was made through transforming CDT11 with the ClpL-luciferase translational fusion amplified from LRC3 using KLO_201 and KLO_206.

### 2.3. Antibiotic Growth Curves

*S. pneumoniae* strains were grown in THY to an OD_600_ 0.2, then diluted 1:100 in 96 well plates containing antibiotics. Ninety-six well plates contained either amoxicillin, cefdinir, or vancomycin at a maximum concentration of 1 μg/mL or 10 μg/mL for vancomycin. Antibiotics were diluted 1:3 with no antibiotic or bacteria controls added. Inoculated plates were read at OD_600_ in a BioTek Synergy HTX BioTek Instruments, Inc., Winooski, VT, USA plate reader every ten minutes for 16 h at 37 °C. The average OD for replicates was calculated, and background signal was removed. MIC was calculated using Prism (v11) using the Gompertz equation following the protocol detailed in Lambert and Pearson (2000) [29]. In brief, the MIC was defined as the concentration corresponding to the inflection point of the fitted Gompertz curve, representing the transition between bacterial growth and inhibition. Goodness-of-fit was assessed by the coefficient of determination (R^2^) and visual inspection of residuals. MIC values are reported as the mean ± standard deviation from at least three independent biological experiments, each containing technical replicate wells.

### 2.4. ClpL Gene Expression

LRC3 and LRC4 were grown in THY and the 96-well-plate setup as described in antibiotic growth curve methods with some variations. THY used in the experiment contained 340 µg/mL of luciferin (Thermo Fisher Scientific, Waltham, MA, USA). The plate reader measured OD_600_ and RLU at a 5 min interval. Luminescence was standardized by dividing the RLU value with the corresponding OD_600_ value.

### 2.5. Antibiotic Susceptibility of Inducible ClpL

LRC2 was grown in THY and the 96-well-plate setup and analyzed, as described in antibiotic growth curve methods with some variations. Antibiotics were added to the plate as described above, with the addition of IPTG at a max concentration of 256 µM followed by a 1:1 dilution in triplicate for each antibiotic concentration tested. Dilution was made across two 96-well plates, and the final three columns had no IPTG added.

### 2.6. PBP2x Surface Exposure

Detection of HiBiT tagged PBP2x was done in a 96-well-plate reader following the protocol described in antibiotic susceptibility of inducible ClpL, with some modifications. Media used for HiBiT tag detection followed the manufacturer’s protocol for Nano-Glo HiBiT Extracellular Detection (Promega Corporation, Madison, WI, USA). In brief, THY is diluted 1:1 with complete detection reagent, which includes buffer, 1:100 dilution of LgBiT fragment, and 1:50 Nano-Glo substrate. LRC2 was diluted to an OD_600_ of 0.1 in 96-well plates, and luminescence and OD_600_ read in the plate reader (BioTek Synergy HTX) every 5 min for 2 h.

### 2.7. Cell Wall Cross-Linking

The *S. pneumoniae* strains MNZ41 (WT) and JLB01 (MNZ41 Δ*aliD*Δ*aliC*) were grown in THY to an OD_600_ of 0.05. Strains were pelleted and suspended in a 50 mM sodium phosphate buffer containing 200 µM HADA followed by 10 min incubation at 37 °C. The strain was then fixed with 2% glutaraldehyde in 0.1 M of phosphate buffer for 15 min at room temperature. The washed strain was then stained with BODIPY FL vancomycin (2 µg/mL) for 5 min at 37 °C followed by washing and fixed again as above. Cells were then visualized on a Nikon C2 scanning confocal microscope. (Nikon Instruments Inc., Melville, NY, USA) Quantification of fluorescence intensity from each stain allows for estimation of the relative proportion of cross-linked versus newly synthesized peptidoglycan, providing insight into cell wall alterations induced by *aliD*Δ*aliC* deletions. The MicrobeJ plugin was used to calculate HADA and vancomycin fluorescence for each cell, and the ratio of the signal was calculated.

## 3. Results

### 3.1. Antibiotic Susceptibility Due to the Presence of aliD

**Figure 1:**
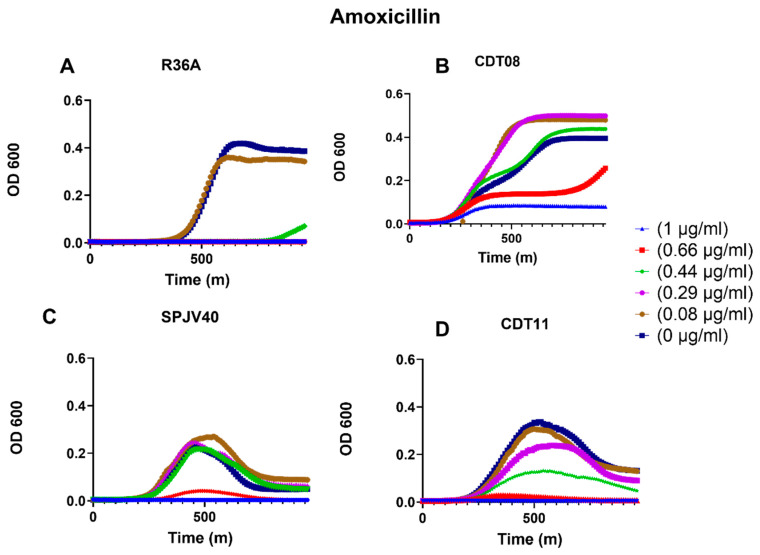
Growth curves comparing susceptibility to amoxicillin with or without the oligopeptide transporter *aliD*. R36A, a serotype two capsule mutant naturally deficient in *aliD* grown in the presence of amoxicillin (**A**), expresses reduced fitness compared to R36A with genetically added *aliD*, CDT08 (**B**). Serotype 38 *aliD*-expressing WT strain SPJV40 (**C**) grown in the presence of beta lactam amoxicillin with concentrations ranging from 1 to 0 µg/mL, expressing greater fitness than SPJV40 *aliD* mutant CDT11 (**D**).

**Figure 2:**
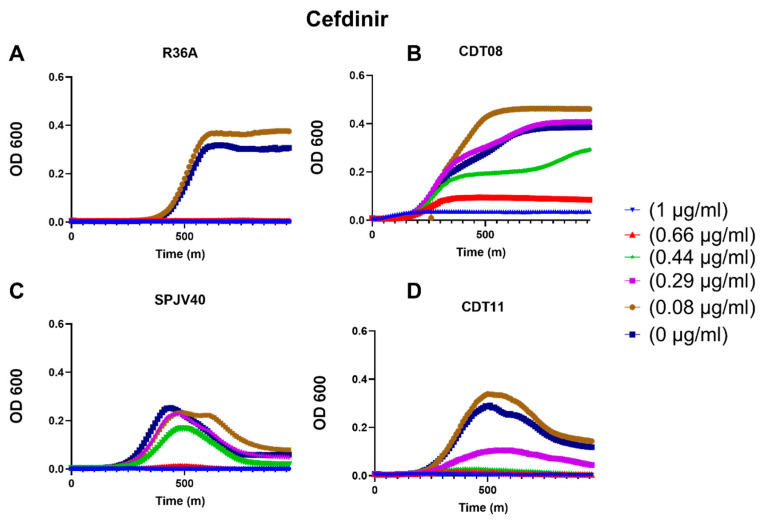
Growth curves comparing susceptibility to cefdinir with or without the oligopeptide transporter *aliD*. R36A (**A**) serotype two capsule mutant naturally deficient in *aliD* grown in the presence of cefdinir, expresses reduced fitness compared to R36A with genetically added *aliD,* CDT08 (**B**). Serotype 38 *aliD*-expressing WT strain SPJV40 (**C**) grown in the presence of beta lactam cefdinir with concentrations ranging from 1 to 0 µg/mL, expressing greater fitness than SPJV40 *aliD* mutant CDT11 (**D**).

First, we wanted to determine if gene regulation by *aliD* impacts antibiotic resistance. To do this, we examined how growth is impacted in the presence of different antibiotics with and without the oligopeptide transporter *aliD*. The strains R36A, which does not natively express *aliD*, and CDT08 (R36A::*aliD*) were grown in the presence of amoxicillin (Figure 1A,B). Compared to R36A, we observed increased growth of CDT08 at all antibiotic concentrations tested, with R36A growth only observed at the lowest concentration (0.08 μg/mL). A more modest difference was noted between SPJV40 (WT serotype 38 with *aliD*) and CDT11 (SPJV40Δ*aliD*), with growth of WT occurring at 0.44 μg/mL compared to minimal growth at the same concentration when *aliD* is deleted in CDT11 (Figure 1C,D). Similar to growth in the presence of amoxicillin, CDT08 exhibited enhanced growth at higher cefdinir concentrations compared to its *aliD*-deficient parental strain R36A. CDT11 (SPJV40Δ*aliD*) showed no growth at either 0.44 μg/mL or 1.0 μg/mL, while the parental strain SPJV40 still grew at 0.44 μg/mL (Figure 2C,D). These observations suggest that the presence of *aliD* may confer a growth advantage under β-lactam antibiotic stress, indicating a potential role in β-lactam sensitivity.

Furthermore, calculation of cefdinir MIC values shows that SPJV40 has an MIC of 0.5909 ± 0.1 µg/mL, and CDT11 presents an MIC of 0.3666 ± 0.09 µg/mL; this implies that *aliD* has an effect on the MIC of β-lactams. This remains consistent with the strains R36A having an MIC of 0.2237 ± 0.06 µg/mL and CDT08 having an MIC of 1.017 ± 0.09 µg/mL (Table 3).

**Figure 3:**
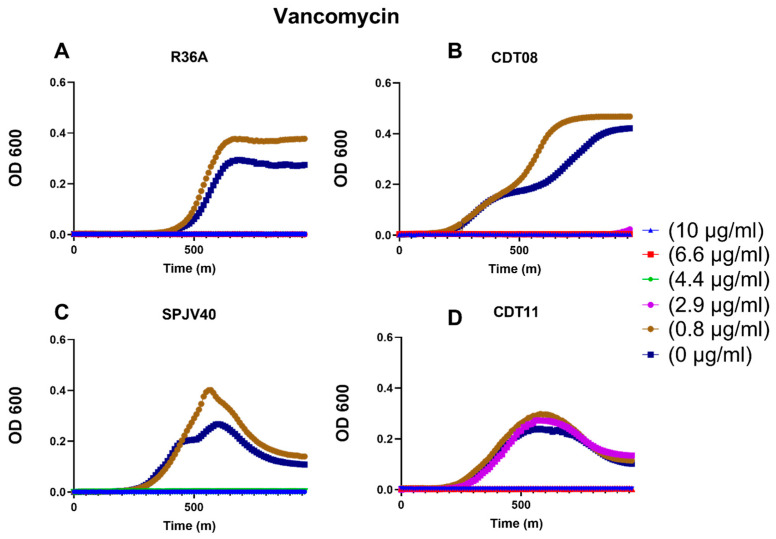
Growth curves comparing susceptibility to vancomycin with or without the oligopeptide transporter *aliD*. R36A (**A**) serotype two capsule mutant naturally deficient in *aliD* grown in the presence of vancomycin, expresses reduced fitness compared to R36A with genetically added *aliD* CDT08 (**B**). Serotype 38 *aliD*-expressing WT strain SPJV40 (**C**) grown in the presence of antibiotic vancomycin with concentrations ranging from 10 to 0 µg/mL, expressing greater fitness than SPJV40 *aliD* mutant CDT11 (**D**).

Next, we wanted to examine if other antibiotics that target the cell wall would also change the growth rates based on *aliD* expression. We tested growth in vancomycin, which targets the cell wall but has a different mechanism of action than the β-lactam antibiotics initially tested. We observed no notable change between CDT08 and R36A growth in the presence of vancomycin (Figure 3A,B). Surprisingly, a difference was observed between CDT11 and SPJV40, with the *aliD* deletion seemingly having increased fitness to vancomycin exposure (Figure 3C,D).

**Figure 4:**
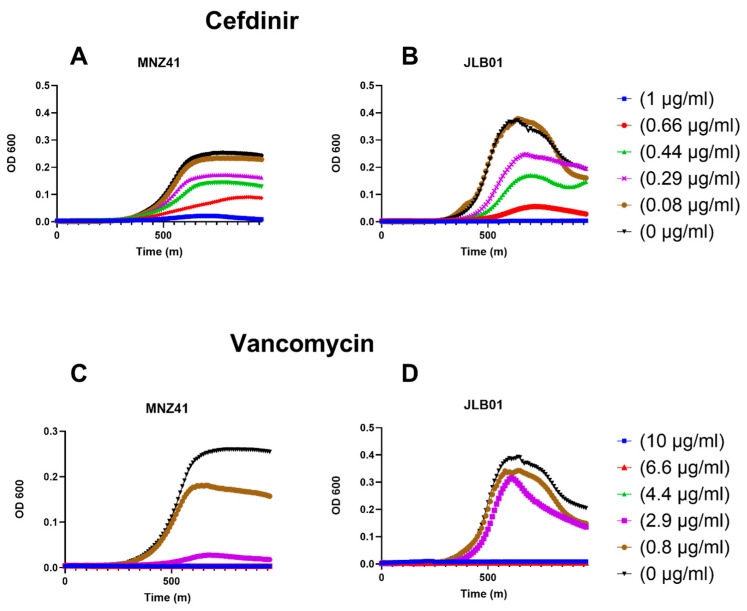
Growth curves comparing susceptibility to vancomycin and cefdinir with or without the oligopeptide transporter *aliD* in nonencapsulated pneumococcus. NESp *aliD*-expressing WT strain MNZ41 (**A**) grown in the presence of beta lactam cefdinir with concentrations ranging from 1 to 0 µg/mL, expressing greater fitness than MNZ41 *aliD* mutant JLB01 (**B**). JLB01 (**D**) grown in the presence of vancomycin, grown in concentrations ranging from 10 to 0 µg/mL, expresses reduced fitness compared to MNZ41 with WT *aliD* (**C**).

In conjunction, we also examined the effect *aliD* has on NESp resistance. *aliD* was first characterized from NESp strains, so the WT MNZ41 and JLB01 (MNZ41Δ*aliC*Δ*aliD*) growth was tested in the presence of both cefdinir and vancomycin (Figure 4). A similar trend was observed when examining NESp antibiotic susceptibility, with WT MNZ41 displaying increased growth in the presence of cefdinir than the isogenic mutant JLB01.

Examining growth in these different antibiotics indicates that *aliD* impacts susceptibility to β-lactam antibiotics. To determine a possible mechanism explaining this difference in susceptibility, available transcriptomic and proteomic data was examined. β-lactam antibiotics specifically target PBPs, while vancomycin targets peptidoglycan subunits [10,22]. Of the genes regulated by *aliD* that may impact PBP activity, the gene *clpL* is a likely candidate for a possible mechanism. ClpL has been previously shown to mediate PBP2x transport to the surface. PBP2x is a transpeptidase that facilitates the creation of cross-links between peptidoglycan strands, and alterations in its expression can impact the structure and stability of the cell wall.

### 3.2. Changes in clpL Expression Due to aliD

**Figure 5:**
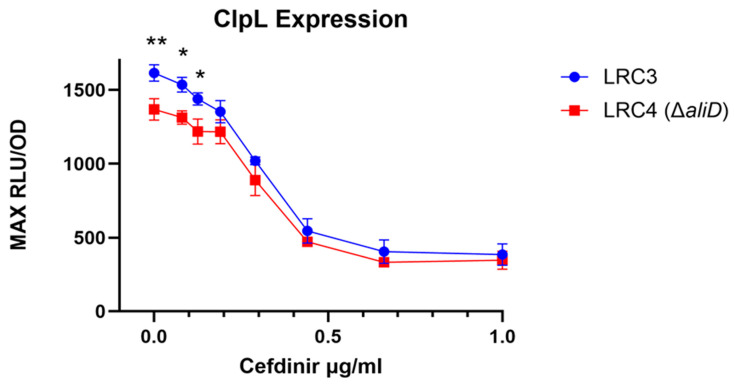
ClpL expression compared via luminescence based on the presence of *aliD* and antibiotics. LRC3 (SPJV40::*clpL*-luciferase) is a serotype 38 construct with *clpL* expression measured through luciferase activity. LRC4 is an *aliD* deletion of LRC3. Both strains were grown in increasing concentration of the β-lactam cefdinir. We observed that *aliD* results in more *clpL* expression in our zero concentration and in the presence of cefdinir (* = *p* < 0.05, ** = *p* < 0.005).

We wanted to further characterize the relationship between *clpL* expression and *aliD*. To test this, a transcriptional fusion of *clpL* and a luciferase reporter were created in the *aliD* containing SPJV40 (LRC3) and its mutant lacking *aliD* (LRC4). This allows for quantification of *clpL* expression based on luminescence intensity. With no antibiotics present, we observed a standardized luminescence value of ~1614 RLU in LRC3 and a significantly lower (~20%) *clpL* expression in LRC4, suggesting the presence of *aliD* increases *clpL* expression (*p* = 0.0075, Figure 5). Furthermore, with the addition of cefdinir, this trend is consistent at 0, 0.08, and 0.125 µg/mL, with LRC3 exhibiting significantly higher standardized RLU than LRC4 (Figure 5). No difference in *clpL* expression is observed at higher antibiotic concentrations, likely due to reduced bacterial growth in these conditions.

### 3.3. Amoxicillin Susceptibility Due to clpL Expression

**Figure 6:**
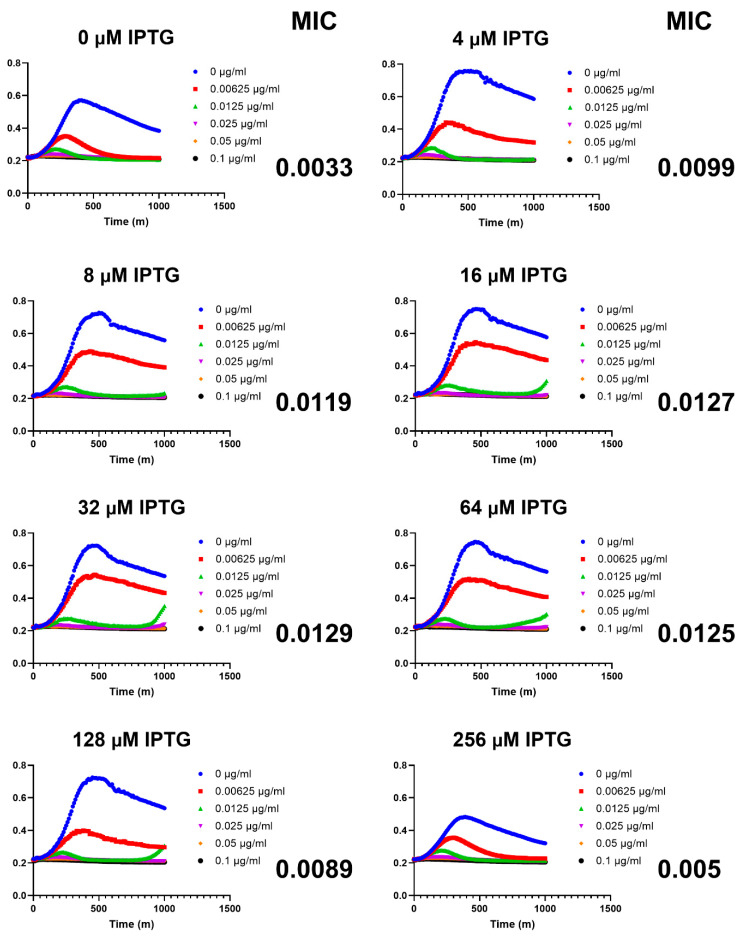
Antibiotic susceptibility to amoxicillin under varying levels of *clpL* expression. Growth kinetics of the LRC1 strain were assessed under varying levels of *clpL* induction controlled by IPTG introduction in varying levels of antibiotic exposure. Antibiotic susceptibility was quantified by calculating the MIC using the Gompertz growth model fit to the area under the curve acquired from optical density data.

To further validate that changes in *clpL* expression impact β-lactam susceptibility, an inducible *clpL* expression system was created in the susceptible R36A background (LRC1). Induction of *clpL* with 4 µM IPTG resulted in a threefold increase in MIC to amoxicillin compared to the uninduced condition. Further increases in IPTG concentration led to incremental MIC elevation, reaching a plateau between 32 and 64 µM IPTG. Notably, at 256 µM IPTG, bacterial growth was impaired, likely due to proteotoxic stress associated with overexpression of *clpL*, a component of the caseinolytic protease (Clp) system (Figure 6).

### 3.4. Surface Exposure of PBP2x

**Figure 7:**
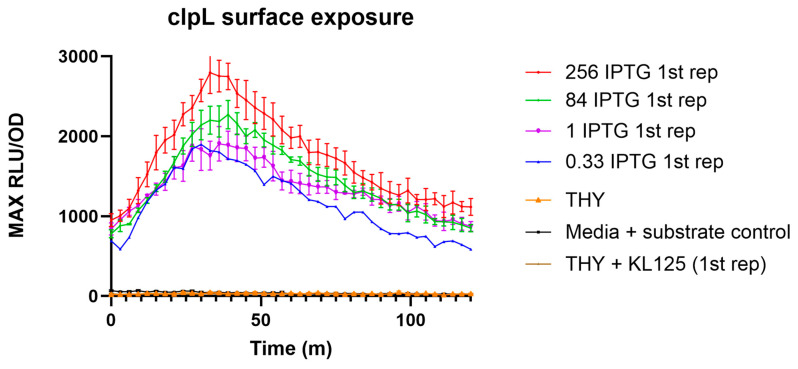
Measurement of PBP2x on the cell wall following increased levels of *clpL* expression. PBP2x contains a HiBiT tag that luminesces in the presence of LgBiT protein fragment and substrate. Increased induction of *clpL* through IPTG addition led to greater amounts of PBP2x observed on the bacterial surface, with 256 µM IPTG induction leading to the highest surface exposure of PBP2x. Intermediate levels of PBP2x are observed as IPTG concentration is reduced.

Since ClpL transports PBP2x to the bacterial surface, which in turn alters cell wall stability and antibiotic susceptibility, we wanted to determine the amount of PBP2x on the cell surface. To do this, we engineered PBP2x with a HiBiT tag under native expression in the LRC1 (R36A::*lacI*; Δ*clpL*; *P_lac_*-*clpL*) background, creating LRC2. Upon addition of the LgBiT fragment and the luciferase substrate, the amount of surface-exposed PBP2x can be determined through luminescence intensity. We observed an increase in luminescence based on the quantity of IPTG-inducing *clpL* expression with our high concentrations of 256 µM IPTG expressing a maximum luminescence of 2795, while our concentration of 0.33 µM IPTG had a luminescence of only 1898 (Figure 7). This indicates that, by increasing the amount of ClpL, a corresponding increase in surface-exposed PBP2x occurs.

### 3.5. Quantification of Cell Wall Cross-Linking

**Figure 8:**
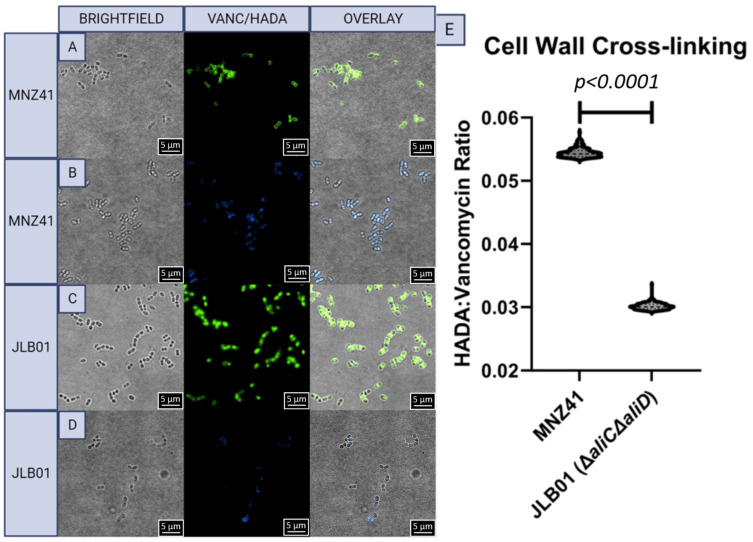
Quantification of cell wall cross-linking due to the presence of *aliD*. The *S. pneumoniae* strains MNZ41 (WT) (**A**,**B**) and JLB01 (MNZ41 Δ*aliC*Δ*aliD*) (**C**,**D**) were labeled with fluorescent vancomycin (green) to monitor the amount of new cell wall formed, and HADA (blue) indicates the quantity of cross-links created. Fluorescent intensity in the blue and green channels was measured for each cell, and the ratio to the total new cell wall and cross-linking per cell was calculated. This ratio is displayed in a violin plot, which indicates significantly higher cross-linking occurring when *aliD* is present. (**E**) Cell wall cross-linking.

Since PBP2x mediates cross-linking of the cell wall, we wanted to examine if there is a correlation between the presence of *aliD* and cell wall cross-linking. To test this, we used two fluorescent dyes to compare the amount of new cell wall that is being synthesized to the amount of cross-linking between these segments. The newly synthesized cell wall was quantified using fluorescent vancomycin, and cross-linking was determined through incorporation of the fluorescent HADA probe. These two dyes allow us to quantify the ratio of newly created cell wall to the amount of cross-linking. Comparison of WT *S. pneumoniae* strain MNZ41 to a mutant lacking *aliC* and *aliD* (JLB01) demonstrated a significantly higher ratio of new cell wall to cross-linking in MNZ41 than the mutant JLB01, *p* < 0.0001 (Figure 8).

## 4. Discussion

Bacteria utilize multiple mechanisms to respond to antibiotic stress, and here we demonstrate that the oligopeptide transporter *aliD* alters antibiotic susceptibility to β-lactam antibiotics through regulation of *clpL* expression. Growth curve analysis revealed measurable differences between tested strains, suggesting that the presence of *aliD* is associated with decreased susceptibility to β-lactam antibiotics. In the antibiotic growth curves, we also observed no growth differences following treatment with vancomycin, which is significant because vancomycin targets the D-Ala-D-Ala terminus of peptidoglycan rather than penicillin-binding proteins (PBPs) recognized by β-lactam antibiotics. This suggests that the observed phenotype is limited to β-lactam antibiotics and the functioning of PBPs. We demonstrate that *aliD* increases *clpL* expression and PBP2x recruitment to the surface. This allows for increased cross-linking in the cell wall and is the likely cause of the reduced β-lactam susceptibility.

Prior work examining the role of *aliD* in virulence used transcriptomic and proteomic data to identify 42 genes regulated by *aliD* [23]. Many of these genes are associated with virulence, carbohydrate metabolism, or stress responses. Among these genes, *clpL* was of particular interest as a possible gene influencing β-lactam susceptibility. ClpL is a chaperone that transports misfolded proteins to the Clp protease system. ClpL is also a chaperone that assists in the folding, stability, and transport of PBP2x. This second function of ClpL suggests a role in cell wall structure, and other published work has shown that *clpL* deletions result in altered cell wall structure and increased stress sensitivity [18]. While all pneumococcal strains express *clpL*, the *aliD* gene is usually found in the capsular polysaccharide synthesis locus of nonencapsulated strains. However, there are three pneumococcal serotypes that express *aliD*: 25A, 25F, and 38. For this study, encapsulated strains expressing *aliD* were prioritized for investigation due to the clinical relevance of encapsulated pneumococci, which are responsible for the majority of pneumococcal disease. This is particularly important in the context of the emerging serotype 38, which has shown increased prevalence and may possess a selective advantage due to *aliD*-mediated peptide sensing and regulation of virulence-associated genes [25].

To more directly link ClpL to antibiotic resistance, we engineered a construct that enables controlled *clpL* expression and tested growth across increasing concentrations of β-lactam antibiotics. We observed that increased *clpL* expression correlated with improved growth under antibiotic stress, indicating decreased susceptibility. However, this positive effect plateaued and reversed at high ClpL levels, where bacterial growth was impaired. This suggests that while moderate *clpL* expression improves protein handling and cell wall synthesis under stress, excessive ClpL may be detrimental due to dysregulation of protease and chaperone systems. This highlights the importance of proper regulation of proteostasis systems for normal bacterial growth and cell wall synthesis.

Since ClpL is a chaperone, we next examined how *clpL* expression affects the amount of PBP2x present on the cell surface. Using an HiBiT-tagged PBP2x, we observed that increased *clpL* expression produced a corresponding increase in luminescence signal, indicating greater surface abundance of PBP2x. Interestingly, induction of *clpL* with 256 µM IPTG had the highest amount of PBP2x transported to the surface but reduced overall pneumococcal growth. Therefore, the amount of PBP2x is the limiting factor to the amount of PBP2x able to be transported to the surface. This supports the model that ClpL promotes PBP2x surface localization or stability, which in turn increases cell wall cross-linking and reduces susceptibility to β-lactam antibiotics. With increased *clpL* expression regulated by *aliD*, if other factors increase the expression of PBP2x, even more cross-linking in the cell wall could occur, resulting in a more pronounced difference in β-lactam susceptibility.

We were able to demonstrate that the presence of *aliD* increases *clpL* expression and PBP2x recruitment to the cell surface. PBPs perform partially redundant functions but produce different types and amounts of peptidoglycan cross-links [26]. Changes in the relative abundance of specific PBPs can significantly impact cell wall structure. PBP1a is typically the most highly expressed PBP, followed by PBP2x, and shifts in the ratio of these proteins can alter cell wall cross-link structure and impact antibiotic susceptibility [27].

To verify that *aliD* impacts cell wall structure, we used fluorescent vancomycin and HADA to measure new cell wall synthesis and cross-link formation. Due to the increased presence of PBP2x on the cell surface, increases in cross linking were expected. Vancomycin labeling was used alongside HADA as a normalization method to account for differences in peptidoglycan synthesis. Using this approach, we determined that deletion of *aliD* in a NESp substantially reduced cross-link formation relative to total new cell wall production, indicating that *aliD* contributes to efficient cell wall cross-linking activity.

This study details a novel role of the oligopeptide transporter *aliD*. Oligopeptide transporters are important regulators that influence gene expression based on the peptide pools available in the local environment. This suggests that the type of infection may influence antibiotic susceptibility, with certain tissues increasing *clpL* expression more than other tissues. The differences in peptide compositions could alter *aliD* signaling and downstream gene regulation of additional genes and impact virulence variability based on the tissue type. This may have important clinical implications, as MIC values determined in laboratory media may not accurately reflect antibiotic susceptibility in the human body, where peptide availability differs substantially from standard laboratory growth media. Therefore, peptide availability and environmental conditions may play an underappreciated role in regulating antibiotic susceptibility in *S. pneumoniae*.

The Ami transporter has previously been shown to decrease susceptibility to various antibiotics by acting as a pore for antibiotic removal [30]. This is unlikely to affect the mechanism we propose in this paper, as *aliD* is located on the cell wall but further demonstrates the need to increase research on the role of oligopeptide transporters on antibiotic resistance. Clinical susceptibility testing is dependent on bacterial growth, either on agar or using broth microdilutions. Each of these will have different peptide pools available, and growth in dilutions a single step above can change an intermediate resistance strain to susceptible. This can lead to treatment that may not be as effective as other antibiotic choices. This is particularly important for meningitis treatment since the difference from susceptible to resistance is a small change in MIC. Along with this, not all clinics will use the same method for quantifying resistance, which can impact not only treatment but reporting of resistance patterns in pneumococcal strains across different geographical areas.

Overall, this study advances our understanding of how *aliD* and *clpL* contribute to β-lactam resistance, likely through effects on cell wall synthesis machinery, protein handling, and PBP2x localization. Understanding the interactions regulating cell wall synthesis can help us learn how to better and more accurately treat pathogens such as the pneumococcus with antibiotics. This research could be used to better inform a physician on the choice of treatment based on whether the pneumococcal strain expresses *aliD*.

## 5. Conclusions

We were able to demonstrate that β-lactam susceptibility decreased when *aliD* was present in multiple pneumococcal strains. Along with this, *clpL* expression is reduced in an *aliD* mutant strain, and increased expression of *clpL* decreases amoxicillin susceptibility. Furthermore, by increasing *clpL* expression, the amount of PBP2x on the bacterial surface increases, likely resulting in increased peptidoglycan cross-linking. We were unable to show a direct link between peptide pools imported by *aliD* and regulatory pathways leading to *clpL* expression. Increased intracellular peptide pools could activate proteolytic systems indirectly and not through direct interaction with transcription factors for *clpL*. This remains unresolved and requires further investigation, particularly in clinically relevant encapsulated strains. Another possibility is that the presence of an additional oligopeptide transporter alters the available peptide pool, which can impact peptidoglycan structure [31]. Others have demonstrated in other organisms that altered peptide pools can decrease β-lactam susceptibility by increasing β-lactamase expression [32]. Future work will try and identify a direct link between *aliD*-imported peptides and transcription factors that regulate downstream gene expression and determine function in a broader range of strains [33]. This is of particular importance since the largest change in MIC was observed in the lab strain R36A. With these studies, it would be possible to determine if there is a direct interaction between *aliD* peptides and *clpL* regulation and have a better understanding of resistance mechanisms.

## Figures and Tables

**Table 1:** Construct list.

Strain	Description	Antibiotic Marker	Reference
SPJV40	Serotype 38	N/A	[22]
CDT11	SPJV40Δ*aliD*	Erm (0.3 µg/mL)	[23]
R36A	D39 capsule mutant	N/A	[24]
CDT08	R36A::*aliD*	Kan (300 µg/mL)	[25]
LRC1	R36A::prs1-lacI-tetR;ClpL-deletion;CIL::Plac-*clpL*	Gent (40 µg/mL), Spec (300 µg/mL), Kan (300 µg/mL)	This Study
LRC2	R36A::prs1-lacI-tetR::ClpL-deletion; Plac_*clpL*::PBP2x-HiBiT	Gent (40 µg/mL), Spec (300 µg/mL), Kan (300 µg/mL), Erm (0.3 µg/mL)	This Study
LRC3	SPJV40; *clpL*-luciferase	Kan (300 µg/mL)	This Study
LRC4	SPJV40Δ*aliD*; *clpL*-luciferase	Kan (300 µg/mL), Erm (0.3 µg/mL)	This Study
LRC5	D39::PBP2x-Hibit	Erm (0.3 µg/mL)	This Study

**Table 2:** Construct Gene list.

Primer Name	Sequence	Region Amplified
KLO_448	GACTCGTGCTCGTAAGTTGG	*prs1* Locus
KLO_449	GGCGATTACCAACAATGGAC	*prs1* Locus
KLO_426	ACGGTGTTGACGGTTTAG	*clpL* Upstream Flank
KLO_427	CAAGCA*GGTCTC*CAGATTCTTTACCTCTTTTTGTTATTTATTATTAC	*clpL* Upstream Flank
KLO_428	CAAGCA*GGTCTC*CATCTTTGGATTTTTGTGAGCTTG	Spec Marker
KLO_429	CAAGCA*GGTCTC*CTGTTCTAGCAAAAAACTGGACG	Spec Marker
KLO_430	CAAGCA*GGTCTC*CAACAGAATTTTGAGGATAAAAAAGAAGG	*clpL* Downstream Flank
KLO_431	AAGAACTCGGCAACCTCA	*clpL* Downstream Flank
KLO_254	CATCTC*GCTCTTC*GCTCGAGAAAGTGTAAGCAATT	PEPY-Plac Linearization
KLO_255	CATCTC*GCTCTTC*GGTTCATTAATTTTCCTCCTTATTTATTTAGATCTCA	PEPY-Plac Linearization
KLO_256	CATCTC*GCTCTTC*GAACAACAATTTTAATAATTTTAACAACATG	*clpL* gene
KLO_257	CATCTC*GCTCTTC*GGAGTTAGACTTTCTCACGAATAACCA	*clpL* gene
KLO_201	TTGGGATGACCCTCCTTG	*pbp2x* Upstream Flank
KLO_202	TAAGGG*CACCTGC*TCGTACGCCTTTTGCTGCTGCTTCGCCGCCTGAGCCTGAGCCGTCTCCTAAAGTTAATGTAATTTTTTTAAT	*pbp2x* Upstream Flank
KLO_203	TAAGGG*CACCTGC*ATCGGCGTGACCGGCTACCGGCTGTTCGAGGAGATTCTGTAAAGGAGGAAAATTAATGAACAAAAATAT	Erm Marker
KLO_204	TAAGGG*CACCTGC*ATCGCTAAATTATTTCCTCCCGTTAAATAATAGAT	Erm Marker
KLO_205	TAAGGG*CACCTGC*AACGTTAGGAGACTAATATGTTTATTTCCAT	*pbp2x* Downstream Flank
KLO_206	ATTGTGTATTTAAACACAAAAACACC	*pbp2x* Downstream Flank
KLO_362	ACGTCA*CACCTGC*TCCAACAGCCGCCAGCCGCTCACGCCTTTTGCTGCTGCTTC	
KLO_363	ACGTCA*CACCTGC*GTTGCTGTTCAAGAAGATTAGCTAAAGGAGGAAAATTAATGAACAAAAATATAAAA	
KLO_280	CACGCATTGCAGAATTGG	*clpL* Upstream Flank
KLO_281	GAAGGT*CGTCTC*CTTAGACTTTCTCACGAATAACCA	*clpL* Upstream Flank
KLO_282	GAAGGT*CGTCTC*CCTAAAAGGAGGAATAATGAGATCCG	Luciferase Gene
KLO_283	GAAGGT*CGTCTC*CCTTTACAATTTGGGCTTTCCG	Luciferase Gene
KLO_284	GAAGGT*CGTCTC*GAAAGGAGGAAAATTAATGAACAAAAATAT	Kan Marker
KLO_285	GAAGGT*CGTCTC*GGTCTTATTTCCTCCCGTTAAATAATAGAT	Kan Marker
KLO_286	GAAGGT*CGTCTC*GAGACAGAATTTTGAGGATAAAAAAGA	*clpL* Downstream Flank
KLO_287	TCAAATGAAATCCTGATTGCA	*clpL* Downstream Flank

**Table 3:** MIC calculations.

Strain	*aliD*	Antibiotic	MIC μg/mL	Standard Dev
R36A	−	Amoxicillin	0.1742	0.0214
CDT08	+	Amoxicillin	1.012	0.3093
SPJV40	+	Amoxicillin	0.2625	0.4413
CDT11	−	Amoxicillin	0.314	0.3065
R36A	−	Cefdinir	0.2237	0.0569
CDT08	+	Cefdinir	1.017	0.7257
SPJV40	+	Cefdinir	0.5909	0.1035
CDT11	−	Cefdinir	0.3666	0.0892
R36A	−	Vancomycin	0.2487	0.0383
CDT08	+	Vancomycin	0.258	0.0336
SPJV40	+	Vancomycin	0.207	0.01
CDT11	−	Vancomycin	0.3465	0.0477

## Data Availability

No datasets were created during this study, but raw data is available upon request.
